# The Active Site of a Carbohydrate Esterase Displays Divergent Catalytic and Noncatalytic Binding Functions

**DOI:** 10.1371/journal.pbio.1000071

**Published:** 2009-03-31

**Authors:** Cedric Montanier, Victoria A Money, Virginia M. R Pires, James E Flint, Benedita A Pinheiro, Arun Goyal, José A. M Prates, Atsushi Izumi, Henrik Stålbrand, Carl Morland, Alan Cartmell, Katarina Kolenova, Evangelos Topakas, Eleanor J Dodson, David N Bolam, Gideon J Davies, Carlos M. G. A Fontes, Harry J Gilbert

**Affiliations:** 1 Institute for Cell and Molecular Biosciences, Newcastle University, The Medical School, Newcastle upon Tyne, United Kingdom; 2 York Structural Biology Laboratory, Department of Chemistry, University of York, Heslington, York, United Kingdom; 3 CIISA - Faculdade de Medicina Veterinária, Universidade Técnica de Lisboa, Avenida da Universidade Técnica, Lisboa, Portugal; 4 Department of Biochemistry, Center for Chemistry and Chemical Engineering, Lund University, Lund, Sweden; Stanford University, United States of America

## Abstract

Multifunctional proteins, which play a critical role in many biological processes, have typically evolved through the recruitment of different domains that have the required functional diversity. Thus the different activities displayed by these proteins are mediated by spatially distinct domains, consistent with the specific chemical requirements of each activity. Indeed, current evolutionary theory argues that the colocalization of diverse activities within an enzyme is likely to be a rare event, because it would compromise the existing activity of the protein. In contrast to this view, a potential example of multifunctional recruitment into a single protein domain is provided by *Ct*Cel5C-CE2, which contains an N-terminal module that displays cellulase activity and a C-terminal module, *Ct*CE2, which exhibits a noncatalytic cellulose-binding function but also shares sequence identity with the CE2 family of esterases. Here we show that, unlike other CE2 members, the *Ct*CE2 domain displays divergent catalytic esterase and noncatalytic carbohydrate binding functions. Intriguingly, these diverse activities are housed within the same site on the protein. Thus, a critical component of the active site of *Ct*CE2, the catalytic Ser-His dyad, in harness with inserted aromatic residues, confers noncatalytic binding to cellulose whilst the active site of the domain retains its esterase activity. *Ct*CE2 catalyses deacetylation of noncellulosic plant structural polysaccharides to deprotect these substrates for attack by other enzymes. Yet it also acts as a cellulose-binding domain, which promotes the activity of the appended cellulase on recalcitrant substrates. The CE2 family encapsulates the requirement for multiple activities by biocatalysts that attack challenging macromolecular substrates, including the grafting of a second, powerful and discrete noncatalytic binding functionality into the active site of an enzyme. This article provides a rare example of “gene sharing,” where the introduction of a second functionality into the active site of an enzyme does not compromise the original activity of the biocatalyst.

## Author Summary

Proteins that display multiple activities have typically evolved through the recruitment of different domains, each of which has a specific function. Thus, in a multifunctional protein, the different activities are mediated by spatially distinct domains such that a single domain can provide the specific chemical requirements for one activity. Indeed, current evolutionary theory argues that the colocalization of diverse activities within a single-domain enzyme is likely to be a rare event, as it would compromise the existing activity of the protein when a new function evolves. Nonetheless, a potential example of multifunctional recruitment into a single protein domain is provided by an enzyme that contains a cellulase enzyme module and a discrete noncatalytic cellulose-binding module. In this article, we show that the cellulose-binding module displays esterase activity and that these diverse activities are housed within the same site on the protein. Structural analysis of the enzyme reveals that its catalytic residues also contribute to the noncatalytic cellulose-binding function. This report provides a rare example of “gene sharing,” whereby the introduction of a second functionality into the active site of an enzyme does not compromise the original activity of the catalyst.

## Abstract 3

The active of site of an esterase enzyme has acquired a noncatalytic carbohydrate-binding function without compromising its catalytic activity, providing support for the "gene sharing" model of protein diversification.

## Introduction

The different activities displayed by multifunctional proteins are typically domain specific. A natural enzyme system that contains large numbers of proteins with complex molecular architectures is presented by the plant cell-wall–degrading apparatus from a range of microbial species. Plant cell wall degradation, now of great environmental significance, particularly with respect to the generation of renewable and sustainable biofuels [1,2], is a challenging process that requires a large consortium of different enzyme activities. The plant cell wall consists primarily of an array of interlocking polysaccharides. While cellulose, which forms crystalline microfibrils, has a simple chemical structure consisting of β-1,4-linked glucopyranoside moieties, the matrix polysaccharides are chemically complex molecules in which the backbone polymers are protected with both sugars and organic esters [3]. Plant cell-wall–degrading systems thus feature glycoside hydrolases, which cleave the glycosidic bonds that link the sugars, and esterases that remove the diverse acylations [4,5]. In addition to their chemical complexity, plant cell walls have a physical structure that presents a physical barrier to enzyme attack. To compensate for the accessibility problem, plant cell-wall–degrading enzymes generally contain a noncatalytic carbohydrate binding function that, by bringing the biocatalyst into prolonged and intimate contact with its substrate, increases the rate of catalysis [6]. In general these diverse catalytic and noncatalytic carbohydrate-binding activities are housed in discrete modules within the same protein [7].

**Figure 1:**
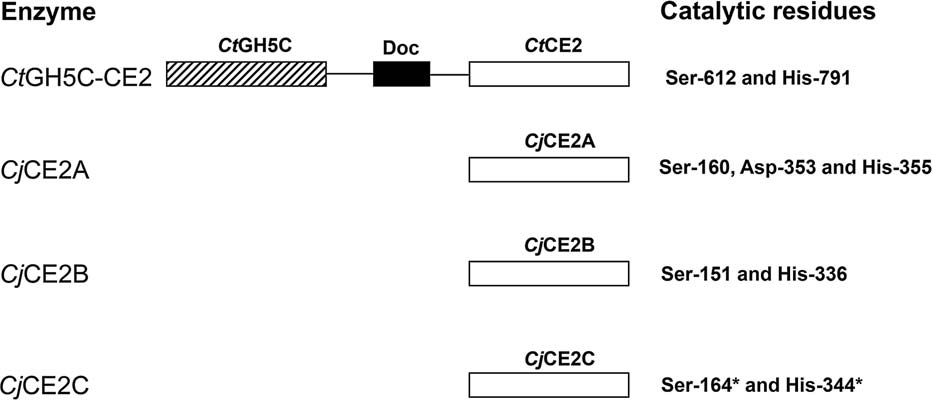
Schematic of the Molecular Architecture of the Enzymes Investigated in This Study The mature forms of the C. japonicus esterases consist of a single catalytic module. The C. thermocellum enzyme containing a CE2 module (*Ct*Cel5C-CE2) consists of an N-terminal GH5 cellulase module (*Ct*Cel5C), a central type I dockerin module (Doc) that facilitates the integration of the enzyme into the cellulosome, and a C-terminal CE2 module (*Ct*CE2). The CE2 modules of all the esterases contains an N-terminal domain of ∼160 residues that displays a jelly roll fold and a C-terminal domain that exhibits an α/β-hydrolase fold. The black lines are ∼15-residue P/T linker sequences. The residues in *Cj*CE2C that carry an asterisk are predicted catalytic residues based on sequence alignments.

A family of carbohydrate esterases (family CE2 in the CAZy classification (http://www.cazy.org/ [7,8]) are especially intriguing with respect to the multiple functions required of plant cell-wall–degrading systems. CE2 enzymes are acetyl esterases (that are generally not appended to other catalytic modules), which are reported to be active on synthetic aryl-esters and acetylated xylan [9]. The bacterium Clostridium thermocellum contains a single CE2 member, designated *Ct*CE2, which is linked to the cellulase, *Ct*Cel5C, within the modular protein designated *Ct*Cel5C-CE2. The enzyme also contains a type I dockerin module that, by binding to cohesin modules in the scaffoldin protein, incorporates *Ct*Cel5C-CE2 into the multienzyme plant cell-wall–degrading complex known as the cellulosome [10] (Figure 1). Intriguingly, *Ct*CE2 was previously characterized as a carbohydrate-binding module (CBM) by virtue of its cellulose-binding capacity and its ability to potentiate the cellulase activity of the linked *Ct*Cel5C catalytic module [11,12]. This unusual, potentially dual, activity of the *Ct*CE2 module prompted us to investigate the functional nuances within the CE2 family.

Here we report the biochemical properties and crystal structure of several CE2 members, both single-module CE2 enzymes and *Ct*CE2, a component of *Ct*Cel5C-CE2. The data show that CE2 enzymes are α/β-hydrolases in which *Ct*CE2 displays a unique dual function within the same region of the protein scaffold. The enzyme module displays acetyl esterase activity that is spatially coupled to a noncatalytic cellulose-binding function. The binding ability directs the appended cellulase module *Ct*Cel5C to, and facilitates its activity on, cellulose. Structural and biochemical analyses reveal that the grafting of aromatic residues into the substrate binding cleft of the enzyme, primarily in harness with His-791, a critical component of the Ser-His catalytic apparatus, also plays an important role in the cellulose-binding function. By contrast, the single-module CE2s, although exhibiting substantial structural homology and similar catalytic activities to the *Clostridium* esterase, do not bind cellulose. We demonstrate how subtle modifications in the active centres of different members of an enzyme family leads to functional divergence, which is manifested by the gain of dual function within the same environment of the protein.

## Results

### CE2 Is a Large Family of Diverse Esterases Displaying the α/β-Hydrolase Fold with a Serine Nucleophile

To probe the function and role of the diverse members of the CE2 esterase family, which currently contains 31 members (http://www.cazy.org/fam/CE2.html), the biochemical properties of five CE2 enzymes were assessed. In addition to *Ct*CE2, which comprises the C-terminal region of *C. thermocellum Ct*Cel5C-CE2 (formerly EGE; [11,12]) we also characterised three Cellvibrio japonicus CE2 members, *Cj*CE2A, B and C and the Bacteroides thetaiotaomicron esterase *Bt*CE2, identified from the genome sequence of the two bacteria [13,14]; these latter four enzymes are not appended to other enzyme modules (Figure 1). All of these CE2 enzymes act as acetyl esterases, releasing acetate from activated artificial substrates such as 4-nitrophenyl acetate (4-NPAc; see Table 1) and, to different extents, the acetylated plant polysaccharides xylan and glucomannan, Table 2 (note that only very small amounts of the *Bacteroides* CE2 could be produced and so only initial qualitative assays could be carried out with this enzyme). Based on their catalytic efficiencies, *Ct*CE2 and *Cj*CE2B exhibit a significant preference for acetylated glucomannan over xylan, whereas *Cj*CE2A and *Cj*CE2C do not distinguish between the two polysaccharides. It should be noted that the specificity of *Ct*CE2 for glucomannan reflects an extremely low *K*_M_. The esterases appear specific for acetyl groups and do not hydrolyse aryl-ferulates or aryl-coumarates (unpublished data). The only other reported analysis of the catalytic activity of CE2 enzymes are the esterases from Neocallimastix patriciarum [9] that display xylan esterase activity and hydrolyse 4-NPAc, but their activity on other substrates was not assessed. The activities reported here extend the substrates known to be deacetylated by CE2 enzymes

**Table 1:**
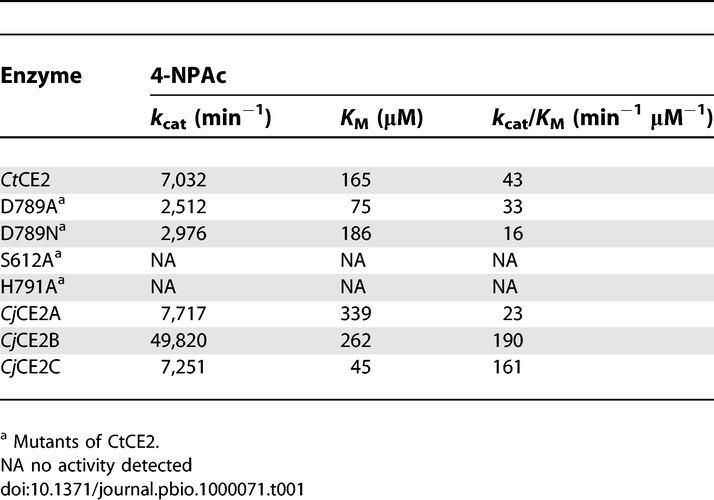
Catalytic Activity of CE2 Enzymes against 4-Nitrophenyl Acetate (4-NPAc)

**Table 2:**
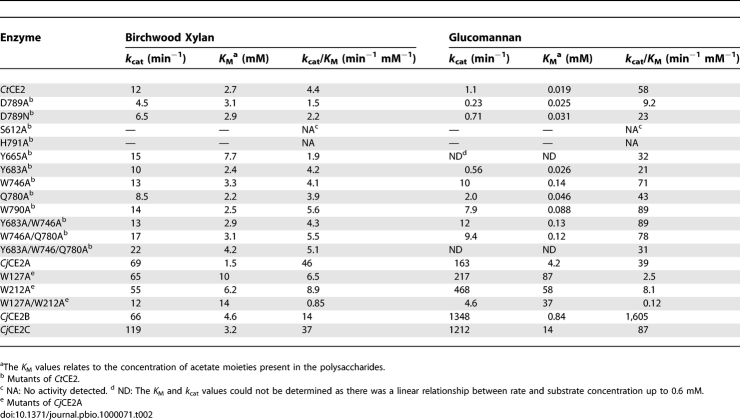
Activity of CE2 Esterases against Acetylated Polysaccharides

**Figure 2:**
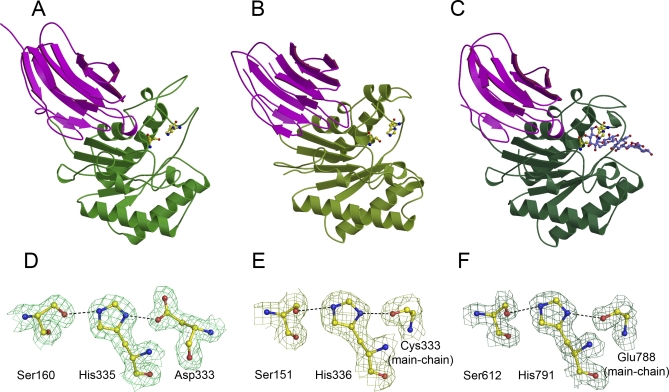
3-D Structures and Catalytic Constellation Geometries for Three CE2 Esterases (A) 3-D structure of *Cj*CE2B with the catalytic domain in green and the β-sheet domain in magenta. The catalytic Ser and His are shown in ball-and-stick representation. (B) *Cj*CE2A, drawn as above. (C) *Ct*CE2 as above and with cellopentaose in blue. (D) The catalytic Ser-His-Asp triad of *Cj*CE2A. (E) The catalytic Ser-His dyad with the main-chain carbonyl interaction from Cys333 of *Cj*CE2B. (F) The catalytic dyad and main-chain carbonyl of *Ct*CE2. This figure was drawn with PyMOL (DeLano Scientific, http://pymol.sourceforge.net/).

For three of these enzymes, we were able to obtain 3D crystal structures (Figure 2A–2C). These include the CE2 module of *Ct*Cel5C-CE2 and two of the *Cellvibrio* enzymes, *Cj*CE2A and *Cj*CE2B. All three 3D structures reveal a bi-domain enzyme in which an N-terminal β-sheet “jelly roll” domain (around 130 residues) is linked to a C-terminal domain of approximately 220 residues. The C-terminal domains possess an atypical α/β-hydrolase (SGNH-hydrolase) fold [15], consisting of repeating β-α-β motifs that form a curved central five-stranded parallel β-sheet, in the strand order β2, β1, β3, β4, and β5 with strand β2 interrupted by loop insertion. The sheet packs against two α-helices (α1 and α6 on the concave side and three α-helices, α2, α4, and α5, on the convex side), all of which are antiparallel to the β-strands. There is also a small α-helix (α3 in the loop connecting β3 and α4 and a 3_10_ helix between β1 and α1. Superimposition of *Ct*CE2 with the two *Cellvibrio* esterases reveals that both the N-terminal β-sheet domain (root mean square deviation [rmsd] of 1.5 and 1.9 over 107 and 94 Cα atoms of *Cj*CE2A and *Cj*CE2B, respectively) and the C-terminal catalytic domain (rsmd of 1.3 and 1.4 over 196 and 195 Cα atoms of *Cj*CE2A and *Cj*CE2B, respectively) display considerable structural conservation.

Structural similarity searches using secondary-structure mapping [16] indicate that the C-terminal α/β-hydrolase domain contains the esterase catalytic centre in which, to date, a Ser-His dyad is invariant across the whole CE2 landscape. *Cj*CE2A is a canonical serine hydrolase, with a classical Ser-His-Asp triad, in which Ser-160 is the catalytic nucleophile, His-335 activates the serine, and Asp-333 makes a hydrogen bond with Nδ1 of His-335 thus completing the catalytic triad (Figure 2D). The oxyanion hole, which stabilises the incipient tetrahedral transition-state, comprise the N of Ser-160 and Gly-205 and the Nδ2 of Asn-255. Indeed, in the pocket within the cleft of *Cj*CE2A is a formate molecule, mimicking the reactive intermediate, which makes hydrogen bonds with the residues that form the oxyanion hole.

In the case of *Ct*CE2 the putative catalytic dyad is provided by Ser-612 and His-791 consistent with the observation that the mutants S612A and H791A display no esterase activity, Tables 1 and 2. In this enzyme the oxyanion hole, similar to *Cj*CE2A, comprise the N of Ser-612 and Gly-658 and the Nδ2 of Asn-705 with formate again mimicking the tetrahedral transition state. *Ct*CE2 along with *Cj*CE2B does not possess a side-chain residue equivalent to the Asp of a classical Ser-His-Asp triad. Instead, these enzymes display a catalytic dyad with stabilisation of the histidine provided by main-chain carbonyl groups (Figure 2E and 2F). *Ct*CE2 does possess an aspartate whose carboxylate group is approximately 5–6 Å from the His-791 Nɛ1, which could, conceivably and with considerable conformation change, make an appropriate interaction with the imidazole ring. In order to probe this possibility, the D789A and D789N mutants were constructed but both retain significant catalytic activity against 4-NPAc and the polymeric substrates (Tables 1 and 2). Instead of the more typical Ser-His-Asp, the “triad” geometry is completed through the Nɛ1 of His-791 making a hydrogen bond with the backbone carbonyl of Glu-788, in the case of *Ct*CE2 (Figure 2F), whilst the equivalent interaction in *Cj*CE2B is with the main-chain carbonyl of Cys-333 (Figure 2E). Catalytic dyads, although rare, have been observed in different forms elsewhere. Other than the CE2 enzymes reported here, IroE is an α/β-hydrolase peptidase that also features a catalytic Ser-His dyad [17]. Given that current wisdom suggests that the Asp of the triad does not act as a base [18], both IroH and now CE2 highlight the need for correct orientation of histidine, which may, apparently, equally well be achieved through an interaction with a carbonyl moiety rather than a carboxylic acid.

**Figure 3:**
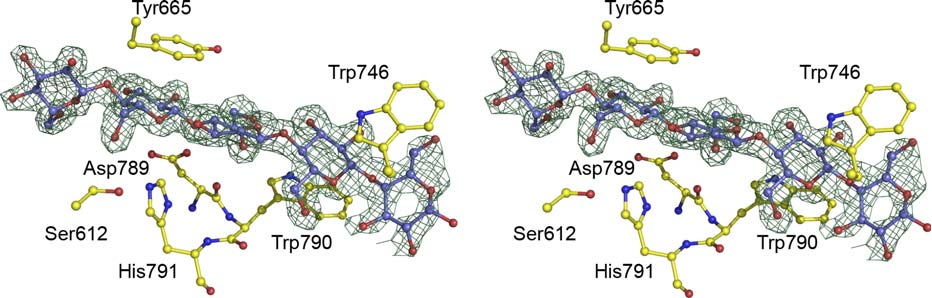
Binding of Cellooligosaccharides through the Esterase Active Centre of *Ct*CE2 Observed electron density (maximum-likelihood weighted 2F_obs_ − F_calc_ contoured at 1 σ) for cellohexaose (in which cellopentaose is ordered) bound to wild-type *Ct*CE2. Ser-612 and His-791 form the catalytic dyad, with Trp-790 causing a change in position of Asp-789 and forming the binding-platform for the second glucose. Other interactions with aromatic residues discussed in the text are shown. The figure is in divergent (“wall-eyed”) stereo and was drawn with PyMOL.

The unusual divergence to a dyad geometry in CE2 likely reflects the inclusion of a loop modification incorporating a tryptophan residue. In *Ct*CE2, for example, one of the key residues involved in cellulose recognition is Trp-790 in the sequence Asp-Trp-His. Inclusion of the Trp forces the adjacent aspartate into a conformation that prevents it hydrogen-bonding with His-791 (Figure 3). The dyad geometry and the topology of the active-centre binding surfaces are described below in light of the dissection of polysaccharide recognition in CE2 enzymes. Sequence analysis suggests that the dyad geometry is not unique to these two enzymes, with 14 of the 26 CE2 members lacking the putative catalytic aspartate. Furthermore, of the remaining 12 CE2 enzymes that appear to have a canonical catalytic triad, 10 have a tryptophan between the His and Asp, as observed with *Ct*CE2, suggesting that in these enzymes the aromatic ring may also place the Asp into an orientation that prevents it forming a classical triad.

### The Esterase and Cellulose-Binding Functions of *Ct*CE2 Are in Close Proximity

**Figure 4:**
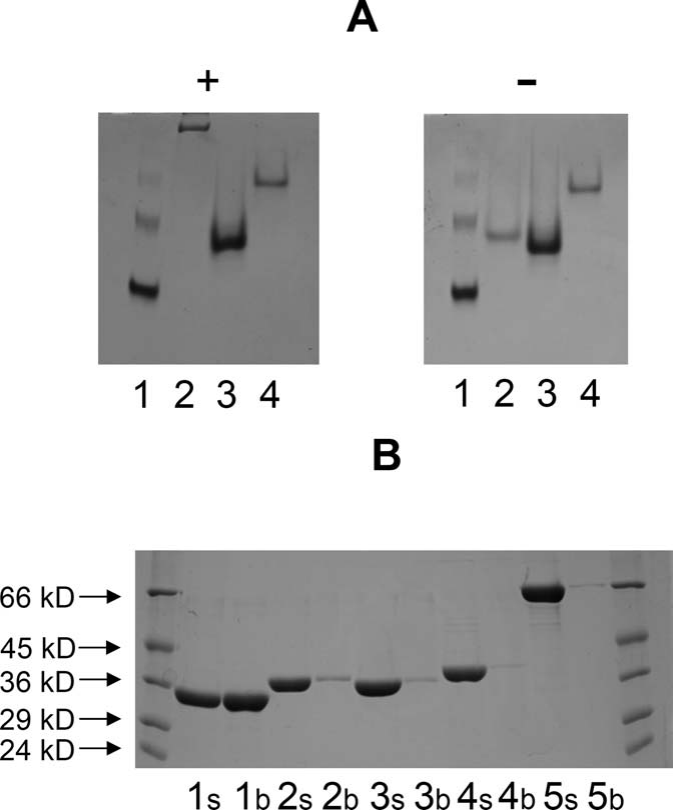
Binding of CE2 Esterases to Cellulose (A) An AGE experiment in which the enzymes were subjected to nondenaturing gel electrophoresis in the absence (−) or presence (+) of 0.1 % (w/v) hydroxyethylcellulose. The lanes contained BSA (1), *Ct*CE2 (2), *Cj*CE2A (3), and *Cj*CE2C (4). *Cj*CE2B did not migrate on the nondenaturing gel. (B) Shows a pull-down experiment using insoluble cellulose. The original protein samples (s) and bound protein eluted from cellulose with 10% SDS (b) were subjected to SDS-PAGE. The lanes contained *Ct*CE2 (1), *Cj*CE2A (2), *Cj*CE2B (3), *Cj*CE2C, (4), and BSA as a noninteracting control (5). Molecular weight markers are shown.

**Figure 5:**
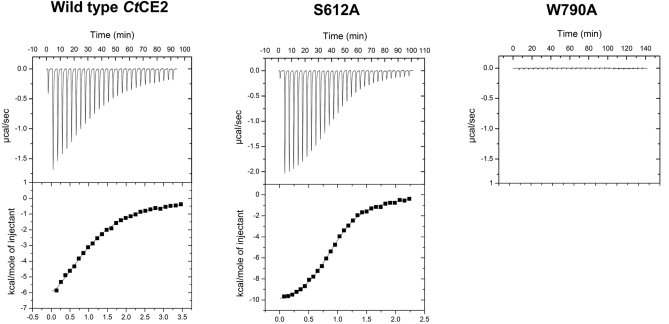
Examples of Isothermal Titration Calorimetry of Wild-Type and Mutants of *Ct*CE2 The proteins were titrated with cellohexaose in 50 mM sodium HEPES buffer, pH 7.0, at 25 °C. The protein concentration for each titration was 100 μM.

The most intriguing CE2 member is *Ct*CE2, which is derived from the multidomain cellulase/esterase *Ct*Cel5C-CE2. Historical work had shown that *Ct*CE2 not only bound cellulose but also potentiated the activity of the cellulase catalytic module on insoluble substrates [12]: the CE2 module thus behaved as a classical CBM [19]. In this report we show that *Ct*CE2 binds to insoluble cellulose (Figure 4). Affinity gel electrophoresis (AGE) also demonstrated that the protein interacts with β-glucan (β-1,3:β-1,4 mixed linked glucan) and soluble derivatized forms of cellulose (carboxymethylcellulose and hydroxyethylcellulose; Figure 4), but displays no affinity for laminarin (β-1,3-linked glucan), α-linked glucans or the β-1,4-linked xylose polymer, xylan (unpublished data). Isothermal titration calorimetry (ITC) revealed that *Ct*CE2 binds to cellooligosaccharides with a *K*_D_ for cellohexaose of 33 μM (Figure 5; Tables 3 and 4), cellopentaose of 71 μM, cellotetraose of 333 μM and cellotriose >1 mM (Table 4), but no binding to mannohexaose or xylohexaose was detected (unpublished data). The *K*_D_ for β-glucan was found to be similar to that for cellohexaose (Table 4).

**Table 3:**
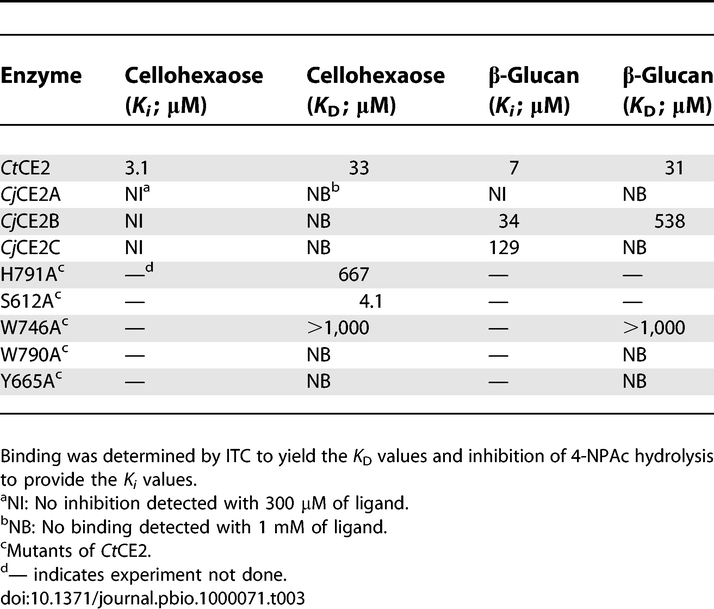
The Binding of CE2 Esterases to Cellohexaose and β-Glucan

**Table 4:**
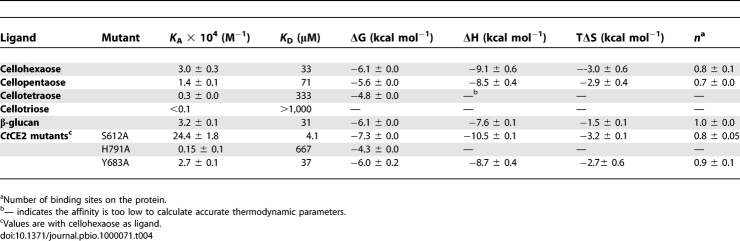
ITC Analysis of *Ct*CE2 Wild-Type and Mutants Binding to Cellooligosaccharides and β-Glucan

Interestingly, whilst all CE2s possess a β-sheet domain that is reminiscent of many CBMs [19], it is not this domain that interacts with cellulose in *Ct*CE2. As was revealed in the 3-D complex structure (Figure 2) and discussed in more detail below, cellooligosaccharide binding, with a stoichiometry of 1, occurs across the esterase active centre with both the Ser and His of the dyad influencing ligand recognition. The S612A mutation of *Ct*CE2 yields an approximately 8-fold increase in affinity (Figure 5; Tables 3 and 4), whilst the H791A amino acid substitution reduces binding with the *K*_D_ increasing approximately 20-fold (Tables 3 and 4). Furthermore, cellohexaose and β-glucan binding inhibit the esterase activity of the wild-type *Ct*CE2 enzyme (Table 3), which is discussed in detail below.

This capacity to bind cellulose is not a common feature within the CE2 family. Pull-down assays revealed that none of the *Cellvibrio* enzymes bound to insoluble cellulose (Figure 4). Furthermore, the C. japonicus CE2 enzymes and the B. thetaiotaomicron esterase were not inhibited by cellohexaose, mannohexaose, or xylohexaose (unpublished data) at 300 μM, while ITC also revealed no binding of the C. japonicus enzymes to these oligosaccharides (Table 3). While the binding of *Cj*CE2A or *Cj*CE2C to polysaccharides could not be detected by ITC (unpublished data) or AGE (Figure 4), *Cj*CE2B interacted with, β-glucan, albeit with ∼17-fold lower affinity than *Ct*CE2 (Table 3), but did not recognize carboxymethylcellulose (unpublished data) or hydroxyethylcellulose (Figure 4). These data reveal that the capacity of *Ct*CE2 to recognise cellulose is, possibly, a unique feature within the CE2 family, and one that prompted its study by X-ray crystallography.

### The Structural Basis for Substrate Recognition in the CE2 Family

The catalytic apparatus of the CE2 esterases is located within a cleft that extends across the catalytic domain and is likely to constitute the substrate binding site of these enzymes. To explore this possibility, the effect of removing the aromatic side chains (which play a pivotal role in protein-carbohydrate recognition [19]) that line the putative substrate binding site (Figure 3) on the catalytic activity of the esterases was investigated. The data, reported in Table 2, show that none of the aromatic residue mutations (Y665A, W746A, and W790A) influenced the kinetic parameters of the esterase against xylan. While the catalytic efficiencies of the *Ct*CE2 variants against glucomannan were similar to the wild-type enzyme, there was a significant increase in *K*_M_, exemplified by the Y665A mutant (*K*_M_ > 0.6 mM), which was mirrored by a similar increase in *k*_cat_. It should also be noted that the activity of the Tyr-665, Trp-790, and Trp-746 mutants of *Ct*CE2 against acetylated glucomannan was subject to substrate inhibition. Thus, these aromatic residues not only bind glucomannan but also guide the polysaccharide into the substrate binding cleft such that the acetyl groups are presented at the active site. These mutational studies indicate that the aromatic residues lining the substrate binding cleft of *Ct*CE2 not only contribute to the tight binding of acetylated glucomannan, reflected by the very low *K*_M_, but also limit oligosaccharide product release after *k*_2_, which appears to be the rate-determining step in catalysis. Mutation of the single aromatic residue in the substrate binding cleft of *Cj*CE2A, Trp-212, although not effecting *k*_cat_, leads to a significant increase in *K*_M_ for both glucomannan and xylan (Table 2). These data suggest that Trp-212 contributes to substrate binding but, in contrast to *Ct*CE2, oligosaccharide product departure at the completion of *k*_2_ is not the rate-limiting step, and thus mutation of the tryptophan does not lead to an increase in *k*_cat_.

In addition to the catalytic α/β-hydrolase domain, the CE2 members also contain an all β-sheet domain, somewhat reminiscent of a CBM. In *Ct*CE2 this jelly-roll domain appears to extend the substrate/cellulose binding cleft of the catalytic domain. To address this issue Tyr-683 and Trp-127, which are located in or at the interface, of the CBM-like domains of *Ct*CE2 and *Cj*CE2A, respectively, were mutated and the activities of the two esterase variants were assessed. The data, reported in Table 2, show that the W127A *Cj*CE2A mutation caused a considerable increase in *K*_M_ but not *k*_cat_ for glucomannan and xylan, while the catalytic properties of the *Ct*CE2 mutant Y683A were similar to the wild-type enzyme. It would appear, therefore, that the CBM-like domain contributes to substrate recognition in *Cj*CE2A, but its role in the catalytic activity of *Ct*CE2 is less evident.

### Structural Characterisation of Cellooligosaccharide Binding to *Ct*CE2

The structure of the α/β-hydrolase domain of *Ct*CE2 revealed a deep cleft. One wall of the cleft is formed by the loops derived from β2, β3, and β4, while the other face contains the extended loop that links β5 to α5. The structure of both wild-type *Ct*CE2 and the S612A mutant were determined in the presence of cellohexaose and clear electron density for five β-1,4-linked d-glucose molecules was observed in the cleft (Figure 3), consistent with the affinities revealed by ITC (Table 4). The interaction of the oligosaccharide with *Ct*CE2 is dominated by planar hydrophobic contacts between the pyranose rings of Glc-1 with Trp-746, Glc-2 and Trp-790, and Glc-4 and Tyr-665 (Figure 3). Hydrophobic interactions represent the major mechanism by which most sugars are recognised by proteins (see for example [20–22]). Although the two structurally characterised *Cellvibrio* esterases do not bind cellohexaose or cellulose, they do contain aromatic residues in the cleft that houses the active site. Thus, Tyr-665 in *Ct*CE2 is conserved in the C. japonicus esterases (Trp-231 in *Cj*CE2A and Tyr-206 in *Cj*CE2B), while Trp-335 in *Cj*CE2B is equivalent to Trp-790 in the *Clostridium* enzyme (Figure S1). The importance of all three hydrophobic interactions in the binding of *Ct*CE2 to cellulose was demonstrated by the observation that the mutants W746A, W790A, and Y665A displayed no, or extremely weak, affinity for cellohexaose or β-glucan (Figure 5; Table 3). Importantly, Trp-746 in *Ct*CE2 is not conserved within CE2 members (Figure S1) (only one other CE2 member appears to contain an aromatic residue at the equivalent position), including the *Cellvibrio* enzymes, suggesting that the insertion of this residue into *Ct*CE2 contributes considerably to cellulose recognition. It should be emphasised, however, that the sequence and conformation of the loop in *Ct*CE2, which contains the critical aromatic residue Trp-746, is different in the other CE2 enzymes. Thus, cellulose recognition is not caused by the simple insertion of a tryptophan into the substrate binding site of the *Clostridium* esterase, but is also influenced by the context of the introduced aromatic residue.

In *Ct*CE2, interaction of cellohexaose with the hydrophobic platform is augmented by several polar contacts (Figure 3). Notably, the O6 of Glc-4 makes a hydrogen bond with Oγ of Ser-612 (in one of its two conformations) in the wild-type esterase and Nɛ2 of His-791 in the S612A mutant, while O6 of Glc-2 interacts with Oɛ1 of Gln-780. As discussed above, mutation of His-791 to alanine reduces affinity for cellohexaose ∼20-fold but, intriguingly, the S612A mutant binds to the oligosaccharide ∼8-fold more tightly than the native protein (Tables 3 and 4). In the wild type enzyme Ser-612 adopts two conformations. In one conformation the hydroxyl makes a polar contact with cellohexaose forcing the ligand away from the histidine. In the S612A mutant the hexasaccharide adopts a different conformation in which it is now able to make a, presumably more productive, hydrogen bond with the histidine. Indeed, the bond distance between Ser-612 and Glc-4 is 3.3 Å (the two conformations adopted by the serine suggests a relatively weak interaction with the glucose), while the bond distance between His-791 and Glc-4 is reduced to 2.8 Å, pointing to a stronger interaction.

It is possible that the CBM-like domain, which appears to abut onto the substrate binding cleft in *Ct*CE2, may also contribute to cellulose recognition. The observation that the Y683A mutant (Tyr-683 is the only aromatic residue in the cleft anterior to the catalytic apparatus) of the esterase retains its capacity to bind cellohexaose (Table 4) and cellulose (unpublished data), however, indicates that the recognition of this polysaccharide is mediated solely by the α/β-hydrolase domain.

### The Mechanism of Cellohexaose Inhibition of *Ct*CE2 Activity

**Figure 6:**
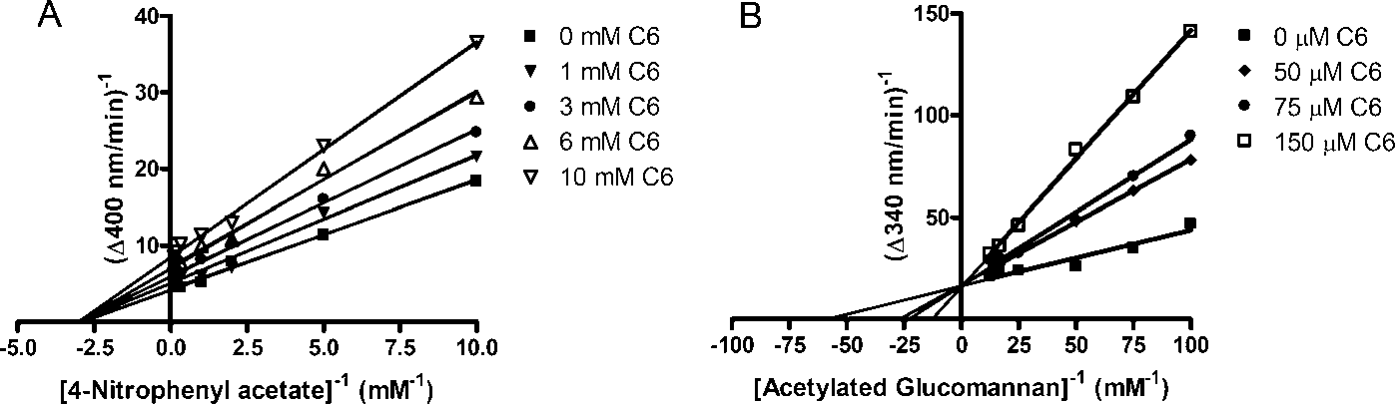
Inhibition of Wild-Type *Ct*CE2 by Cellohexaose CtCE2 was assayed at 37 °C using either 4-nitrophenyl acetate (A) or acetylated glucomannan (B) as the substrate in the presence of different concentrations of cellohexaose (C6). The figure displays double reciprocal plots of the data.

Cellohexaose inhibits the deacetylation of glucomannan by *Ct*CE2 with a *K*_i_ of 32 μM (Figure 6A), similar to the *K*_D_ of the esterase for the hexasaccharide determined by ITC (Table 4). Consistent with its capacity to interact with the catalytic apparatus and with aromatic residues in the substrate binding cleft, cellohexaose is a competitive inhibitor of glucomannan, as indicated by the double reciprocal plots which intersect on the *y*-axis (Figure 6A). In contrast, cellohexaose inhibits the hydrolysis of 4-NPAc by *Ct*CE2 with a *K*_i_ of 3.1 μM, which is ∼10-fold lower than the ITC-determined *K*_D_ of the enzyme for the ligand. In addition, the inhibition by cellohexaose displayed noncompetitive kinetics (Figure 6B: double reciprocal plots intersect on the *x*-axis), suggesting that the ligand inhibits the deacetylation of glucomannan and the aryl-acetate by different mechanisms.

*Ct*CE2, typical of enzymes that mediate catalysis through a covalent intermediate, displays biphasic kinetics against substrates, such as 4-NPAc, which contain good leaving groups. Pre-steady state kinetics, deploying 4-NPAc as the substrate, revealed a *k*_2_ (pre-steady state rate of 4-nitophenolate release) value of ∼21,790 s^−1^ with an amplitude that was ∼80% of the predicted value, while *k*_3_, extracted from the steady state rate, was ∼4,725 s^−1^ (unpublished data). Significantly, cellohexaose inhibited enzyme deacylation (the steady state rate; *k*_3_), with a *K*_i_ of 7 μM, but did not significantly affect enzyme acylation (the pre–steady state burst of 4-nitrophenolate release; *k*_2_).

The data described above are consistent with the observed noncompetitive kinetics displayed by cellohexaose when 4-NPAc is the substrate, and the difference between *K*_i_ and *K*_D_. ITC measures binding to the apoenzyme (available to the substrate at *k*_2_) while *K*_i_ determines affinity of the oligosaccharide for the enzyme at the rate-limiting step of the reaction, *k*_3_, when the enzyme is in complex with acetate. Indeed, the kinetics of inhibition are consistent with the observation that cellohexaose binds more tightly to the enzyme when it makes a polar contact with His-791 rather than Ser-612; when the serine is in complex with acetate (or formate in the crystal structure) it is not available to hydrogen bond with the hexasaccharide that is now able to interact with His-791. By contrast, *k*_2_ is the rate-limiting step when glucomannan is the substrate and thus cellohexaose must now bind to the apo form of the enzyme to inhibit the reaction leading to a lower affinity (as it will make a polar contact with Ser-612 and not His-791) and competitive kinetics.

## Discussion

Family CE2 esterases raise intriguing questions about the optimization of plant cell-wall–degrading systems. The most interesting feature of the divergence of function within the CE2 family is that *Ct*CE2, derived from the *Ct*Cel5C-CE2 multimodular enzyme, is uniquely able to act as a CBM, with structural and biochemical studies highlighting Trp-746, a residue observed in only one other CE2 enzyme, as a key recognition element. It is probably highly relevant that *Ct*CE2 is the only member of CE2 that is a component of a modular enzyme. Thus, *Ct*CE2 is an example of an emerging biochemical theme that protein scaffolds possess a latent potential to acquire new, orthogonal functionality [23,24].

This report is in contrast to the Ohno model of protein evolution, which proposes that mutations that provide new functionalities are introduced into a redundant copy of a duplicated gene [25,26]. A central component of the Ohno model is that the introduction of new functions into a protein compromises its original activity, hence the requirement for gene duplication subsequent to mutation. The observation that the chicken structural protein δ-crystallin and the metabolic enzyme, argininosuccinate lyase, are encoded by the same gene led to the “gene sharing” hypothesis, which states that a gene can be recruited for a novel function without significant changes to its protein coding sequence [27]. Bergthorsson et al. [28] and others argue that Ohno's model poses a dilemma, because it requires that the duplicated copy of the gene, which incurs an energetic cost, must be retained within a population prior to acquiring the mutations that confer the new activity. These concerns led to the proposal that gene products display secondary activities prior to duplication of the encoding gene, and that there is a selection for both activities subsequent to amplification (these models are reviewed in [29]). A major caveat with models based on “gene sharing,” however, is that amino acid changes that introduce new functions often compromise the stability or original activity of the protein [30]. Thus, in general, the introduced functionality replaces the endogenous activity of the enzyme. Here we show that the active site of *Ct*CE2 displays dual activities; it catalyses the deacetylation of plant polysaccharides and also potentiates the activity of its appended cellulase catalytic module through its noncatalytic cellulose binding function. As such, *Ct*CE2 provides an example of gene sharing in which the new function (cellulose binding) has developed without having an obvious deleterious effect on the existing esterase activity of the enzyme. It would appear, therefore, that there are different ways to evolve new enzyme functions without compromising the original activity displayed by the protein. Which of these pathways a protein follows depends on (i) what the new function is, (ii) whether it is advantageous to retain both activities, and (iii) whether the chemistry or steric constraints of the two divergent functions preclude the housing of these activities in the same active site.

Throughout plant cell-wall–degrading systems, noncatalytic binding to polysaccharides by enzymes that attack insoluble substrates is primarily conferred by CBMs that are linked to, but spatially distinct from, the catalytic module [19]. In general these targeting modules are joined by flexible linker sequences, although noncatalytic carbohydrate binding regions, while spatially distinct from the active site, can occasionally be distal components of the catalytic module itself [31,32]. This report shows that the presentation of an additional tryptophan residue within the substrate binding cleft of the esterase enables the enzyme to acquire cellulose-binding capacity—potentiating the activity of the appended cellulase catalytic module—whilst still retaining its original catalytic activity. Indeed, the importance of cellulose recognition by *Ct*CE2 in the function of the appended catalytic module *Ct*Cel5C is supported by the observation that mutation of Trp-790, Tyr-665, or Trp-746 in the full-length enzyme (*Ct*Cel5C-CE2) reduces the activity of the cellulase against insoluble substrates by 3- to 5-fold. Hence the CE2 family displays a spectrum of activities reflecting the grafting of new functionality upon the α/β framework. This manifests itself most powerfully in the acquisition of cellulose binding by *Ct*CE2, demonstrating how nature has exploited latent carbohydrate recognition to introduce additional “noncatalytic” polysaccharide binding features that are complementary to the activities displayed by a complex modular enzyme. The composite structure of the plant cell wall, which requires the synergistic interactions of multiple catalytic and binding functions to elicit its degradation, exerts selection pressures that lead to the generation of single proteins with multiple activities. The CE2 family, and *Ct*CE2 in particular, reveal an extreme example of this functional complexity and provides a platform for the future directed engineering of plant cell-wall–degrading enzyme systems, which is one of the key environmental goals of the twenty-first century.

## Materials and Methods

### Gene cloning and protein expression.

The region of the C. thermocellum cellulase–esterase gene, *cel5C-ce2*, encoding the C-terminal CE2 esterase module, *Ct*CE2 (residues 482 to 814 of the full-length enzyme), was amplified by PCR from genomic DNA (strain ATCC 27405) using the thermostable DNA polymerase Pfu Turbo (Stratagene) and primers (Table S1) that contain NdeI and XhoI restriction sites, respectively. The DNA product was cloned into the NdeI and XhoI sites of the Escherichia coli expression vector pET22b (Novagen) to generate pCtCE2. *Ct*CE2 encoded by pCtCE2 contains a C-terminal His_6_ tag. The region of the genes encoding the mature C. japonicus and B. thetaiotaomicron VPI-5482 CE2 esterases were amplified by PCR from genomic DNA, using primers listed in Table S1, and cloned into NdeI- and XhoI-restricted pET22b such that the proteins encoded by the recombinant plasmids contained a C-terminal His_6_ tag.

### Protein expression and purification.

E. coli BL21 cells harbouring the CE2 esterase-encoding recombinant expression vectors were cultured in Luria-Bertani broth at 37 °C to mid-exponential phase (*A*_600nm_ ∼0.6–1.0), and recombinant protein expression was induced by the addition of 1 mM isopropyl 1-thio-β-D-galactopyranoside (IPTG) and incubation for a further 5 h at 37 °C. The CE2 esterases were purified by immobilized metal ion affinity chromatography (IMAC) using Talon resin (Clontech) and elution in 20 mM Tris/HCl buffer containing 300 mM NaCl and 100 mM imidazole. The eluted esterase was then dialyzed against 20 mM Tris/HCl buffer, pH 8.0 (Buffer A) and applied to a Bio-Rad Q12 column. The esterases were eluted with a 400 ml gradient of 0–500 mM NaCl in Buffer A. The fractions containing esterase activity were concentrated using a 10 kDa MWCO Vivaspin 20 centrifugal concentrator and applied to a Superdex 200 26/60 Hiload column (Amersham) equilibrated in 10 mM Tris-HCl buffer, pH 8.0, containing 150 mM NaCl. Protein was eluted at a flow rate of 1 ml/min. Both chromatography steps employed a Bio-Rad FPLC system. Purified enzymes were adjudged homogenous by SDS-PAGE. To produce seleno-methionine–containing proteins the same protocol was employed except that the enzyme was expressed in E. coli B834 (Novagen) using growth conditions described previously [33]), and 2 mM 2-mercaptoethanol was included in all buffers during purification up to the point of IMAC and 10 mM for all subsequent steps. The purified seleno-methionine enzyme eluted from the gel filtration column was exchanged into ddH_2_O containing 10 mM DTT using Amersham PD10 column.

### Mutagenesis.

Site-directed mutagenesis was carried out using the PCR-based QuikChange site-directed mutagenesis kit (Stratagene) according to the manufacturer's instructions, using pCtCE2 or pCjCE2A as the template and primers pairs that are listed in Table S1.

### Enzyme assays.

Substrates used in the enzyme assays described below were purchased from Sigma except acetylated Konjac glucomannan, which was purchased from Megazyme International (Bray, Ireland); acetylated xylan, which was prepared from birchwood xylan (Sigma) using acetic anhydride following the method of Johnson et al. [34], and O-acetyl galactoglucomannan that was extracted from spruce (Picea abies) [35]. To determine activity against 4-NPAc, 1 ml enzyme reactions were carried out in 50 mM sodium phosphate buffer, pH 7.0, containing 1 mg/ml of BSA and substrate concentrations up to 10 mM. The reaction was initiated by the addition of an appropriate concentration of enzyme; 10 nM for wild type and 10 μM for the most inactive mutants, and the release of 4-nitrophenolate was monitored at 400 nm. To determine the rate of deacetylation of acetylated polysaccharides, the release of acetate was determined using an acetic acid detection kit (Megazyme International) following the manufacturer's recommendation except that the product was measured continuously rather than in a stopped reaction. Pre-steady-state kinetics were performed using a stopped-flow apparatus (Applied Photophysics Model SX-17MV), with the flow path thermostatically controlled at 15 °C, and a 2-mm light path to monitor the formation of the product 4-nitrophenolate at *A*_400_. Equal volumes (50 μl) of solutions mixed with a dead time of 1.5 ms, contained, respectively, 10 μM *Ct*CE2 and 2 mM 4-NPAc in 50 mM sodium phosphate buffer, pH 7.0. These solutions were supplemented with 0–100 μM cellohexaose.

### Carbohydrate binding studies.

The capacity of the CE2 enzymes to bind to soluble saccharides was determined by AGE or by ITC. AGE was performed as described previously [33] with the polysaccharide ligand included in the polyacrylamide gel at 0.1 %. ITC [36] was carried out in 50 mM HEPES-Na buffer, pH 8.0, containing 300 mM NaCl at 25 °C. Data collected for titrations with 10 mg/ml of β-glucan were fitted with a molar concentration of 5 mM, the value at which *n* = 1. The concentrations of cellooligosaccharides in the syringe ranged from 0.7 to 5 mM and the CE2 enzymes in the reaction cell were at 80–100 μM. The determined *K_A_* and *ΔH* values were used to calculate *ΔS* from the standard thermodynamic equation. Binding to insoluble cellulose was determined by pull-down assay using phosphoric acid swollen cellulose (PASC). Briefly, 1 mg of washed PASC in 50 mM Tris-HCl, pH 8.0, containing 300 mM NaCl (Buffer B), was mixed with 50 μg of protein in a total volume of 100 μl and incubated on ice for 30 min. This was then centrifuged for 1 min at 13,000*g* and the supernatant containing the unbound protein removed. The cellulose was then washed three times with 100 μl of ice-cold Buffer B and each wash discarded, before 50 μl of SDS-PAGE loading buffer (containing 10 % SDS) was added and the bound protein eluted from the cellulose by boiling for 5 min. Approximately equal volumes of starting protein and material eluted from the cellulose were then subjected to SDS-PAGE to assess binding.

### Crystallisation and structure solution.

*Cj*CE2A: Native and seleno-methionine crystals of *Cj*CE2A were grown by hanging drop vapour-phase diffusion in 1 M diammonium hydrogen phosphate/0.1 M sodium acetate, pH 5.0, for 1–2 d at 20 °C (30 % v/v glycerol was added as the cryoprotectant). Data from seleno-methionine derivatised *Cj*CE2A were collected on ID23–1 at a wavelength of 0.97880 Å using an ADSC Q315R CCD (charge-coupled device) detector the wavelength was optimised for the *f*′′ component of the anomalous signal using a fluorescence scan. Data from crystals of native *Cj*CE2A were collected on ID14.2 using an ADSC Q4 CCD detector at a wavelength of 0.9330 Å. Data for all CE2 enzymes were processed with either DENZO [37] or MOSFLM from the CCP4 suite (Collaborative Computational Project Number 4 1994). The structure of *Cj*CE2A was solved using the single-wavelength anomalous dispersion (SAD) method. Selenium positions were determined using SHELXD [39] and the phases were subsequently calculated using SHELXE. 5% of the data were set aside for cross validation analysis and the behaviour of R_free_ was used to monitor and guide the refinement protocols. ARP/wARP [40] in conjunction with REFMAC [41] was used to automatically build the sequence into the electron density. Refinement was undertaken in REFMAC with manual correction to the model using COOT [42].

*Ct*CE2: Crystals of both wild-type and S612A *Ct*CE2 in complex with cellohexaose were grown by hanging drop vapour-phase diffusion from 20% PEG3350 and 0.2–0.3 M ammonium iodide, with protein at ∼8 mg ml^−1^ and cellohexaose at approximately 1 mM. Crystals were harvested in rayon fibre loops before being bathed in cryoprotectant solution (crystallisation conditions augmented with 25% v/v glycerol) and flash frozen in liquid nitrogen. Data were collected at the ESRF from single crystals at 100 K for cellohexaose complexes of both wild-type and S612A mutant forms with data processed as previously. The structure of *Ct*CE2 was solved by molecular replacement using PHASER with the search model being the *Cj*CE2A structure. ARP/wARP was used to build the initial model and refinement was undertaken as described above.

*Cj*CE2B: Native and seleno-methionine crystals of *Cj*CE2B were grown by hanging drop vapour diffusion in 0.1 M imidazole, pH 8.0, 10 % PEG 8,000, for 3 d at 20 °C (25 % v/v glycerol was added as the cryoprotectant). Native data were collected at ESRF on ID14.2 using ADSC Q4 CCD detector at a wave length of 0.933 Å. The structure was solved using a combined approach in which molecular replacement using a combined *Ct*CE2/*Cj*CE2A model was used in PHASER [43] was used in conjunction with the Se-Met derived SAD phases for map calculation and verification of methionine positions. The final model was build using COOT and refined using REFMAC. *Cj*CE2B crystals contains two molecules in the asymmetric unit. The final models of *Cj*CE2B in chain A was ordered from 27 to 361 containing five missing regions in amino acids 41–44, 61–63, 81–84, 98–98, and 127–137, while chain B was ordered from 26 to 361 and contained one missing region between residues 99 and 107. Coordinates and observed structure factor amplitudes have been deposited at the Worldwide Protein Data Bank (wwPDB, http://www.wwpdb.org/), and the crystal structure statistics are in Table 5.

**Table 5:**
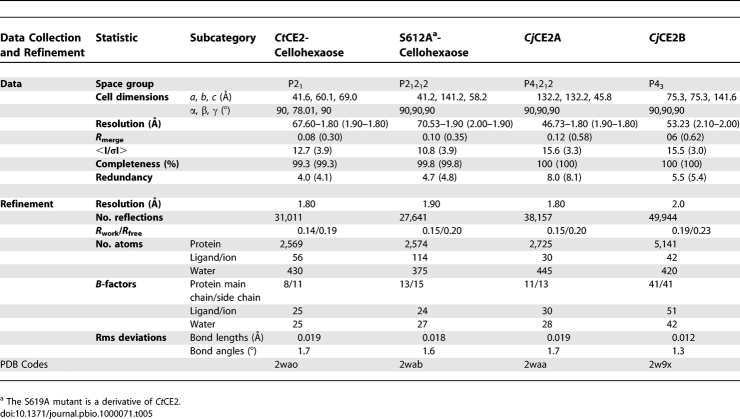
Data Collection and Refinement Statistics

## Supporting Information

Figure S1 Alignment of CE2 Enzymes The catalytic histidine and serine are in red, the residue equivalent to the aspartate that completes the catalytic triad is green, while the equivalent residues to the three aromatic amino acids in *Ct*CE2 that binds to cellulose are blue. The vertical arrow marks the division between the β-jelly roll fold and the α/β-hydrolase catalytic domain. The residues are labelled according to the *Ct*CE2 sequence.
(53 KB DOC)

Table S1 Primers Used to Mutate Residues in *Ct*CE2 or Clone Other CE2 Enzymes (35 KB DOC)

## Abbreviations

4-NPAc: 4-nitrophenyl acetate
AGE: affinity gel electrophoresis
CBM: carbohydrate-binding module
ITC: isothermal titration calorimetry
rsmd: root mean square deviation
