# Supplementary material for: The Active Site of a Carbohydrate Esterase Displays Divergent Catalytic and Noncatalytic Binding Functions
Source: PLoS Biol. 2009 Mar 31;7(3):e1000071. doi: 10.1371/journal.pbio.1000071 (PMC2661963; doi:10.1371/journal.pbio.1000071)
Supplement: Figure S1 — The catalytic histidine and serine are in red, the residue equivalent to the aspartate that completes the catalytic triad is green, while the equivalent residues to the three aromatic amino acids in CtCE2 that binds to cellulose are blue. The vertical arrow marks the division between the β-jelly roll fold and the α/β-hydrolase catalytic domain. The residues are labelled according to the CtCE2 sequence. (53 KB DOC) [file pbio.1000071.sg001.doc]

482

|

***Ct*CE2**  ----------------------------------------------------------DS

***Cj*CE2A** --------------MNTQSL------------------------------------MSST

***Cj*CE2B** --------------------------------------------------------MADS

***Cj*CE2C** ---------------------------MAQAEPAATN-----------------------

ABD82400 --------------MSIQPLRTLVICTAASLICALFSACSTLIP-----------QQAPL

ABF90480 ---------------SVGAGLLVSGCKSASHTPPLAPDAG----------------ALAW

ABD81630 ------------MCLKINRCWVFVWLCICATTAHSET-----------------------

ABG60573 MYTRNVRIPQFQLQMAIALLLSGLIFLSCKKDTVSPSVPIPAV-------------DTNM

ABG58008 MHLCYKSAYMIKKIKSVTCLLVLLMSCMSMETTKAP------------------------

ABF40906 ----------------MISRLLALILALTSFTVAAPPVKFQISP------------VSSS

AAK24425 ------------MRL-----LLSLAAALA----------------------------LST

ABL69400 ----MVMGAGWAKRIGIGMAAIALAAAMAFLAWPAPPRPLTPVPMLSLAEDAGLQAQLVA

ABG62309 ------MGISSVRKLPVAGRLRAVVAAFAAAAFFLGSFP------------------SGA

AAK78516 ------MRKKFSFLLIIFMMLLNVNFTVKAASFG-----------------------YGS

CAN95885 ---------------------------------------------------------AGG

XP_362887 --------------------MAPTQLRFLLHALM-----------------------AAA

ABX41632 ---------------------------MLQRSE---------------------------

AAB69091 ----------TDTVKSVPNDLNESNSENVQNTKSNTT--------------------TAN

ABO90850 --------------MSIALLILWAPFSAAKDVN--------------------------V

AAO07747 -----------MFYRLICSLALGLCTASALAQS--------------------------L

AAO75552 ---------MWKNVLVLVSLCLLFEDVNAQTTKN-------------------------R

ABQ07964 ------------MFLKKRALLILFLLNSIISTS--------------------------Q

ABX41518 --------------MEYNGEITSKVRRGTMGEMR-------------------------K

529

|

***Ct*CE2**  QTPDEDNP------GILYNGRFDFSD----PNGPKCAWSGSNVELNFYGTEASVTI---K

***Cj*CE2A** HTIAASDP------HIQVMGRTHINDD----ASLTFGYPGVSLSTIVAGSRLTAEMQSSN

***Cj*CE2B** TKP----L------PLHIGGRVLVESPANQPVSYTYSWPAVYFETAFKGQSLTLKFDD--

***Cj*CE2C** -LIGAAHA------YYLYTGRVDFSDK----QAPRLSWPGTSIKANFTGTYLAVVLDDEL

ABD82400 QTVAPNHP------MVTVSGRTQTMLD----GSERFGYPGVSFTVNVKAKGVIFNASSTS

ABF90480 VRIPASDA------RVQYLGRTYRSDT----G-VVFSHPGVTIRARFWGDAVRMELHDAG

ABD81630 -YVPADND------QYLYTGRIDFSDI----KAPSLSWPGTSIKANFTGEHLEVVLDDQN

ABG60573 VFIDASNT------NIHYSGRIDFSNP----KAPAFWFPGISIKASFQGPSIDVVIKDDA

ABG58008 CFISIEKF------ILVKRGRYEQTK------E-YISFSNSGTAMGFMAPEEEFYIWIEE

ABF40906 EKTGLHVL------PMHIGGRVLQRG-AESAPSYERQWPGTYFDTAFIGPDVYFKLGS--

AAK24425 PALAQTAL------SLNIGGRVAPAP---NG-AYDFGWPGVYFEGRFTGPSVEIVVDT--

ABL69400 PQPGLAAL------PAAVTGRAAAGP---SGEGYRHEWPGFHASARFEGTGVTLRFDD--

ABG62309 LAQQKLAE------QFRLVGRFVQPS----PSAAIFAWPGSAIEIAFTGSRLDISLRDT-

AAK78516 LKTSETSS------NVLFIGRFDTSD----PAGPKFAWSNSTIKANFNGTGISVNLK---

CAN95885 EDPSDDT--------IRFIGRFDTTN----AEGPRFSWAGSAIVTRFSGTSIGVRFNDES

XP_362887 TVAAEST---------RFLGRVN-------PATRELTWPATGIAVAFDGTTASIPITS--

ABX41632 -WSGDYG-------DVKPIGR-TYS----KDGIRWLALSGSGIEFICETELVEIAIVSDS

AAB69091 LSSGSSNTFQPSKENVKILGRGLYQE---KEGSLWIGLTDSGVEYKFNGKTTTISLTADS

ABO90850 EATDYR---------LSFSGRVVKNWI---NSDVYFNWPGVALSFRFKGMEASIIMNCRG

AAO07747 PATNHN---------LTFEGRNSKNYQ---DGSVAINWPGSTLKTRFIGSHFGLTMQGNG

AAO75552 YYKANDE-------NVSYVGRTEIQS----DGSVSFDWTGTYLRTRLSGGELSMKISDTG

ABQ07964 IQNSAKT--------FLYEGRIDKLQ----NDNVILIGTASSVTFNFTGNECSISLQSVD

ABX41518 VPLEQVEH-------YKVHGRTTKER-----NPLTLFWTGSGIEVNVSGTEIWVELYS-D

580

|

***Ct*CE2**  ----SGGE--NWFQAIVDGNPLPPFSVNATTST--VKLVSGL-AEGAHHLVLWKRTEASL

***Cj*CE2A** -----G-N--SWIDVIIDNHP-PTSIKLDAQQQT-VELFHFP-NSGEHRVEIIHRSENWH

***Cj*CE2B** ------DQ--NIFRLIVDDK--APVVINKPGKV--DYPVESL-APGKHRVRLEKLTETQS

***Cj*CE2C** GKNYFN------IIVDGETRH-PFVLEAKQGEHS-YWISSTL-GEGEHSLEIYKRTEGEE

ABD82400 -----G-N--NYLDVYIDGRL-QRSIKLEKAATD-YVVFNHN-RARQHQVKILNRSESWH

ABF90480 QGGEVG-T--TYFDVVIDGAP-ARQLAVSRHQSS-YLLATGL-EVGLHTVELIKRTESLV

ABD81630 GKNFFN------VIIDGNDRF-PYVLEAKQGEHR-YLISSAL-SKGKHSVEIYKRTEGEE

ABG60573 SGTDQG-T--NYYNIFIDQQL-HTVLKVNAEDTL-YKIARNL-TDANHTIEVFKRTEAQV

ABG58008 TSTNISSC--NWFVVLSDYKQ-IGDIRLKPGKHM-YKIT----APLNSYIQLIKATEAVV

ABF40906 ------GD--AILKITLDQS--S-QSLTKPAPG--LYVIRGL-TNKHHDLRLEVITESQA

AAK24425 ------GG--EHLAVSIDGV--RKAELTKSGQT--RLKLDRL-GPGEHVVRLDKLTESQS

ABL69400 ------SL--NRWRVMLDG---KAVEVSRPGRQ--DLRIEGL-APGPHEIRAEKISEP-S

ABG62309 ------GQ--NSMVVELDGKP-SRLDLRQGKHD--YRVVDGA-PHGPHLLRLIRRTEPMF

AAK78516 ----SSGD--NWFNVIIDGIVKMPVNVTSSNSSP-IVLAEGL-TNGKHTVELVKRTEASV

CAN95885 T---AAQS--NFFQVVIDGEAKGVLKVNSEKEL--YTVAEGL-PDGEHDLVLHRRTEPLV

XP_362887 ----QWGT--VAVEMVIDDG--PPIAIPNVDGSA-ITTPAGL-PRGRHTMTVRKKSEAYF

ABX41632 SVHGQNGTDYARFAMYINEEL-VFCDTLREEQKT-VKIFESI-EKQEITVRILKISEAPH

AAB69091 SAASTDNP--ARIVILADGEL-YEDTTVKSVESE-FEVTFDA-SG-EHTVRFMKVSECAN

ABO90850 ----------GRFDVLVNGRVVSIINTDSTLKQYSLVKFDSQ---QDVVVELVKRNETYE

AAO07747 ----------DYFDVLVDGKLSKTLMTQPNGVTETFVLFTSE-STQNVLIEVVKRTENYA

AAO75552 ---------INYYNVFVDSLLHKTVKVSGKDTL--INFISGM-DKGIHHVLIQKRTEGEW

ABQ07964 S-----YEHHNYVQLVLDGKYIGKIRIEKGAAQS-FPIKVTS-NKKEHRLEIYKNTEAHS

ABX41518 -----YDCFEPWISVLVNGEVISRQMVLKGMHK--LCLFRNMNPDVIKNIQIFKEVQAMP

631

|

***Ct*CE2**  GEVQFLGFDFGSGK--LLAAPKPLERKIEFIGDSITCAYGNEGTSKE-----QSFTP--K

***Cj*CE2A** GQVTLKQLTLTGTQ-FLPAPVLP-QRKILVLGDSVTCGEAIDRVAGE--------DKNTR

***Cj*CE2B** TSGRFLGFYTDPSA-KPLALP-KRKRQIEFIGDSFTVGYGNTSPSRE-----CTDEELFK

***Cj*CE2C** GSTAFKGLVLANGA-RLLLPPERPKRRMEIYGDSISSGMGNEGADNG-------ADHLLS

ABD82400 GLATLHSIRVQDGK-FLAPPAKP-RTKLLVIGDSVTCGTGANRKQGC--------EMDPS

ABF90480 GHSELVALEVHG-E-LREPPARS-GLRMEFVGDSVTCGYGSDVALIPGSPSWTAPTFTSK

ABD81630 GATLFKGLWLADDS-YLLKPPKRPKRRIEIYGDSITSGMGNEGADNG-------ADHLGS

ABG60573 GSSSFKGFCLQANK-NLTASAKP-AHKIEFIGDSYTCGYGNELSIPAPPNGNPDTGFHSV

ABG58008 GEVRIYGLQLHRPY-LQTETILADLKKIQFIGNSITCGYGNMVSVPAPPDGNPLTGFHPA

ABF40906 GPTSFDGFFAPRSA-KPDTPH-SYPLQIEFIGDSHTVGYGNTSPKRE-----CTEDEVWA

AAK24425 GSSRFQGFFVGEGG-KALPAP-ARPRKIEFIGDSHTVGYGSRSTSRS-----CTAQQIHD

ABL69400 GPALFGGFFLGDPA-QALPPPDPVPRLIEFIGDSDTVGFANTAERRD-----CDAEEIYA

ABG62309 GPTVFMGAETDG----SFARPHEKEKSLFVIGDSISAGYGVEGETTS-----CKFS--AD

AAK78516 GEVQFLGFIVDGGE--LISPPQPLQKRIMFIGDSITCGYGNEGKSQY-----QSFTT--K

CAN95885 GVSQFLEFVPEQGE-ALLPVPAAPARRIEVIGDSISAGYGVDGADET-----CPFTS--D

XP_362887 G-SLFVGQPTTDGE--LVDLGPRPKRSIEIIGDSISVGYGLEGVFP------CANAA--D

ABX41632 SMIGIEKIILHSDIP--PKKTEDLAHKIEFIGDSITCGYGVDAESEL-------IHFTTA

AAB69091 GTVRITAIKADAEK---IEPTADADKKIEFIGDSITCAYGVDGTEG---------TFSTK

ABO90850 EMVLVDGLEVNG----EVLEHRTPQKHIIFFGDSLSAGLGSESHHQQ-----CSSDETFK

AAO07747 SMSRFLSVEHNG----SIDGVWGHKPHILFIGDSISAGFGSESEKRE-----CTWEEIYA

AAO75552 GKTTIHQFVLHNEG-ELMKETECPSRHIEFIGNSLTCGYGVEGKDRS-------EPYKAE

ABQ07964 GGILFTGTTAKLT-----AISFKKKKKIEFIGDSITCGAASDPSDVP-----CDKGEYLD

ABX41518 DDEKHCLQVFSFELDGEFYKVPERKAKLEVIGDSITSGEGAIGATCE-------EDWISM

685

|

***Ct*CE2**  NENSYMSYAAITARNLNASANMIAWSGIGLTMNYGG----APGPLIMDRYPYTLPYS--G

***Cj*CE2A** WWNARESYGMLTAKALDAQVQLVCWGGRGLIRSWNG----KTDDANLPDFYQFTLGDTGQ

***Cj*CE2B** TTNSQMAFGPLTAKAFDADYQINASSGFGIVRNYNGT---SPDKSLLSLYPYTLNN---P

***Cj*CE2C** EKNHYWAYGAITARNLNAELHTISQSGIGIMISW-------FPFIMPQFYDQLSAVGN-N

ABD82400 WWDAHNSFGMQLGRALSAETHLVCYGGRGVMRSWNG----EPKDIQAPAFYDLAVPEPWA

ABF90480 NQNPRRTYAWLTAGNLGAEAVLICYSGHGV---YRNLDM-STS-GLVPALYELAVPG--H

ABD81630 EKNNYLAYGAITARNLNAELHTISQSGIGVMVSW-------FPFIMPQFYNQLSAVGN-N

ABG60573 NENNYTAWGAIVARELDAEYHCTAYSGRGI---YRNNNG-AATGVLPSIYNRINPDD--A

ABG58008 NENAYMSYAMQTARKLNADPMLVSYSGKGV---YRNFDG-DTNETLPQIYDRIHLHDK-N

ABF40906 TTDTSQGIAPLVARPFHADYQVNAISGRGIVRNYNG----FPGDTLPAAYPFTLLD---H

AAK24425 LTDTSLAFGPILARRLDADYRIQAFSGRGVVRNYNGG---APGLPLPVLFPRLIPGQE-Q

ABL69400 ATDTSRSFGPQVAAALGADYRIVARSGIGLLRNYGGA---EPDRTMDRLYPLALPG-D-G

ABG62309 TENQYLTYAALVARAFEADVITAAVSGKGLVRNYDG----GAKNTMPEIYLRGLPD----

AAK78516 NENAYLSYGAITSRLLRAEPMTICWSGKGLIRNSGG----NTTDLMPDLYQRILPYTS-T

CAN95885 TENNYLAYSALTGRLLQADTTIVAWSGRGVYRNYNG----EVAPTMPEIYGRTIADEE-Q

XP_362887 NEAATKTYGALTANNLSADYSIVAWSGKGVTRNYVAPGP-DLDPRMPELWTRWGAGDE-T

ABX41632 TEDVTKAYAYLTSKALNSDYSMVSYSGYGIVSGYTENDKKDTIQIVPKYYQSVARCFGTF

AAB69091 TEDGTKSYAYLASKQLNADYSMFSFSGFGIISGYSTDGTRNEVSTVPQYYEKLGFSYWTQ

ABO90850 TSNARVAFPTMTAKLLNASHSQVSYSGLGVIRNYAGE---QRYHNLPYYFNKAGAVLN--

AAO07747 TSNARLAFPYQTGQQLNTTITQVSFSGLGLIRNWGGN---QPHHNLTTYTDKVAAVYG--

AAO75552 TENCNLSYATIIARYFNADYTLIAHSGRGVVRNYGDSVRISAVTMKDRMLNTFDMN---L

ABQ07964 HHNGYYAYGPTLSRAIGAAYLMSSVSGIGMYRNWNDEN--KDEVIMPDAYPNLYLTKDSS

ABX41518 WFSSQDNYATLLAKKLGADLRIISQSGWGVVSSWNN----NPYGAIPPIYEEVCGPLKGE

714

|

***Ct*CE2**  ----------VRWDFSKYVPQVVVINLGTNDF---------------STSFAD------K

***Cj*CE2A** A---------PQWDHHRYQPDLIISAIGTNDF---------------SPG----------

***Cj*CE2B** D---------QLYHNKHWKPQVIVIGLGTNDF---------------STALNDNERWKTR

***Cj*CE2C** D---------SRWNFSQWTPDVVVINLFQNDS---------------WLIDREKKL--QP

ABD82400 D---------APWDNSKFKADIILVSLGTNDF---------------SMG----------

ABF90480 G---------VAWDFSGPSPDVIVVNAGTNDT---------------FAGS--GTD--AY

ABD81630 D---------SIWDFKQWTPHVVVINLMQNDS---------------WLIDREKRL--TP

ABG60573 S---------SQWNTATYIPDVIVIHLGTND-----------------FAPEQLST--PD

ABG58008 S---------LFWDHANQIPDIIVINLGTND-----------------YFGESQNQ--P-

ABF40906 T---------SRYDNPDWRPQVIVVSLGTNDF---------------STPLHAGEKWKTR

AAK24425 P---------RVDVSDSWSPDLLVIGLGTNDF---------------STPLNPGEPWKDE

ABL69400 D---------AVALPQ-RPADIVVVGLGSNVF---------------GSDLAPGEAWRDK

ABG62309 ----------RPGDLPFPRSDVIIVHLGTNDF---------------G--NG-ARP----

AAK78516 ----------PLWDTNRWVPQVVVINLCTNDF---------------SIGIPD-------

CAN95885 ----------PAWDFSSWVPQVVVINLGTNDF---------------SINVPGD-A---Q

XP_362887 ----------GSYDFAAPV-DAVVINLGTNDF---------------SFDPAVRPQ---L

ABX41632 K--DLPLLENIPWDFSKYIPELIVINLGTNDN---------------TYCK-------DK

AAB69091 FGSDITQLKEVSWDASQFVPDLVVINLGTNDN---------------SYMQNVKG---DR

ABO90850 ----------DSSAYEDRHPDLLVIELGLNDF---------------NTDLKPDEPWANV

AAO07747 ----------LTLDYEDKFPNLIVVEVGTNDF---------------STDPQAHEPWSNI

AAO75552 D---------KKWDFKTYKPDLVVVNLGTNDF---------------STNIYP-------

ABQ07964 K---------PKYDFA-FQPDIISIALGTNDF---------------SDGDGKKER---L

ABX41518 RNEALG--AHEKNDFKAWQPDVIVINLGTNDGGAFDQPEWQDERTGETFKERKNEDGTYY

760

|

***Ct*CE2**  ----TKFVTAYKNLISEVRRNYPDAHIFCCVGPMLWG---------TGLDLCRSYVTEVV

***Cj*CE2A** IPDRATYINTYTRFVRTLLDNHPQATIVLTEGAILNGD---------KKAALVSYIGETR

***Cj*CE2B** EALHADYVANYVKFVKQLHSNNARAQFILMNSDQSNG---------EIAEQVG----KVV

***Cj*CE2C** IPDDEQRIQAYIDFVRSIRAQYPKAEIICALG----------SMDATANDKWPDYIKTAV

ABD82400 IPDQQAFINTYVAFATILLRDHPNATIAITDGAILGHDE------LNKKGTLQNYLKQVQ

ABF90480 LPDEAAFKSAYRAFLTRLRTLHPRAHLVCTLGSMSDGRKQLEQNGTTTSAHVGDWLTELV

ABD81630 IPADAQRIAHYQAFVQSIRAEYPKAQIICALG----------SMDATANEKWPNYVREAV

ABG60573 MVDSAAFVSGYINFITTLRSYYPAAKIICVRPNSLTDYY---PVGLKALTRMTRYTKAVT

ABG58008 -LNDTVFVHRYIAFVDRLSTYYPKAQIICANGSMLNDGW---PEGKKCWTRIQENLKKVQ

ABF40906 DELHADYEQTYAEFLHQLRARNPKAYFILWATEMSDG---------EILAEVQ----KVA

AAK24425 AALRKDYRDRYVAFIEALKASRPKAKVFLMAGDSFAE---------DVAEV---------

ABL69400 AELRRDFGPALADFAAARMQENPGAAMVLLAFGEYGP---------ELVEAHR----AAS

ABG62309 ----PGFEERYAAFLEELRKKAPDAMIYAAMGPLLFG---------EDLKAAAGAVKRAV

AAK78516 ---RTTFVTAYSTFIDRIRSQYPTAHIYCAVGPMLNG---------DNLKSARDYINSAV

CAN95885 --FRGPFTEAYAGLVETVRTNYPEAFIFCTIGPMLSDSY---PEGAEALSRARDYIGQVV

XP_362887 --DVDVFSRAFAAFLAQVRGAYPSAVVFLTGSPLLND---------ATAERQKSRHSAVM

ABX41632 TKRHEEYRDEYVKFLKVVREKNMDATILCALGLMG-------------ANLYPMVEEAVA

AAB69091 AKEEADFVADYVKFIEQIRSVHPNAEILCTLGIMG-------------QELYPQIEEAVS

ABO90850 EDFRHSWTDAYVQFIIQLRQRYGDVPIVLVGLELD------------KDDPFERAINAVN

AAO07747 EEVKQAWTERMVEYVSELKHRYHEVPIILMPRPAY------------PYDYIIPATQDAI

AAO75552 --LEDEFIHAYKLLISRLRTNYGDVPILCISPAIA-------------QRQIVQYMERMR

ABQ07964 PFNASKYVSNYINFIKMLYEHNPNVQIVITNSPMVNG---------EKGVVFEECLKKVK

ABX41518 EEDLDRFEWAVRAFLRKLRRLNQNAYLLWVYGILD-------------KPLLPTLQKAIN

814

|

***Ct*CE2**  NDCNRSGDLKVYFVEFP-QQDGSTGYGEDWHPSIATHQLMAERLTAEIKNK------LGW

***Cj*CE2A** QQLHSN-------RVFYASSSHHPGDNSDAHPTKDQHAAMARELTPQLRQIMD-------

***Cj*CE2B** AQLKGGGLHQVEQIVFKGLDYS----GCHWHPSANDDQLLANLLITHLQQKKGI------

***Cj*CE2C** ARMRKDN---KDEKLDTVFFDFTG-YGQ--HPRIAQHKANAEKLTAFIREKMR-------

ABD82400 AAVDSP-------RLAFIPSNIYAGDSCDAHPTGEQHTQMAQDLQVQLEQLLNTNR----

ABF90480 AERQQQGDARVYRHVMAVQNPNVDGVGEDWHPSAATHQKMAEALTWFIRDVVRP------

ABD81630 KNMQ-DN---GDNKIDTIFFEYIG-YGQ--HPRVAQHNANADKLTKFIKKKMKW------

ABG60573 DHFVPT----DANVFYFALAPQNAPYGEDWHPSAATHQSMAAQIKTFIKTRAGW------

ABG58008 EHFQAKG---NTKIYTFFFTPQQGPYGEDFHPSLATHTKMAEELTTFIQTVVNK------

ABF40906 DRVRSAGEKQISVVPVKELEVT----GCNYHPSLTDDRKIADAIVAAIKAKN--------

AAK24425 ---ADRTGAKAVRITGMDLR------ACDWHPSAADQLMMADRLEAAIKSSP--------

ABL69400 ELLAARGLANQLVILGDPRRN-----ACLWHPSAQDHAMIAQTLIEALKGPVPN------

ABG62309 EARAGAGDDKLSFIAFEDPEGREV-RGCDWHPNAAGQEHMADILGARLEADLGWERRE--

AAK78516 EMKNSSGDQKVHFIEFP-VQDSANGYGEDWHPTVKTHELMAVQLYKAIKADLGW------

CAN95885 EDRTADGDDRVRFLEFPPHAKDTDGLGCDWHPSAKKNRDMAQQLAEAIREELDW------

XP_362887 REAAERSGPDVHFVEFPTQDASGNNIGCDYHPSARTNQELAVILTAAMRSVMGWDACKRS

ABX41632 LYQSETKDDNIVTFQLTEQDK-ADGYGADWHPSKVAHQKAAKQLTEEIKKIMGW------

AAB69091 TYKAASGDEKVNAYKFNQQNINKNGKGIDWHPAPQSHVEAAEELVAEIKKLYGW------

ABO90850 EELLRMG-----ERSIYIKKIDIKFDGCLWHPTEVEHKRISTILASFISDTSLLSE----

AAO07747 ETLAQQE-----IHQVYSHTFVSPLEGCIWHPTAQEHREIATQLSQFIRQQALLTTP---

AAO75552 KDLNDKK--VYIAVLPEGLCDSTTDLGAVWHPNYKGQMKMAMSLIPYMSTITGWPLKKES

ABQ07964 NAFAEDK--NHKAIQIFKFKPMTP-KGCTGHPDVADHKVLADEYGPFLKKLLNEK-----

ABX41518 TYQTECEDLKVSLLEIP--KMTEEEIGARYHPGKKAHERMATILSEEIQNIL--------
