# Supplementary material for: The Active Site of a Carbohydrate Esterase Displays Divergent Catalytic and Noncatalytic Binding Functions
Source: PLoS Biol. 2009 Mar 31;7(3):e1000071. doi: 10.1371/journal.pbio.1000071 (PMC2661963; doi:10.1371/journal.pbio.1000071)
Supplement: Table S1 — (35 KB DOC) [file pbio.1000071.st001.doc]

| **Residue mutated/gene cloned** | **Primers** |
| --- | --- |
| *Cj*CE2A | CAGCTCCAGCATATGAATACGCAATCACTTATGTCATCCAC |
| *Cj*CE2A | CTCCAGCTCGAGCCCATTGCGATAGTTACCCAGGAGCCGA |
| *Cj*CE2B | CAGCTCCAGCATATGGCCGACTCAACCAAGCCGC |
| *Cj*CE2B | CTCCAGCTCGAGCCAGATACCTTTTTTCTGCTGCAAATGG |
| *Cj*CE2C | CAGCTCCAGCATATGGCCCAGGCGGAGCCGGC |
| *Cj*CE2C | CTCCAGCTCGAGCCAACGCATTTTTTCCCGGATAAATGC |
| D308A -F | GCACCGGATACGGAGAA**GCG**TGGCATCCAAGTATTGCCACCC |
| D308A -R | GGGTGGCAATACTTGGATGCCA**CGC**TTCTCCGTATCCGGTGC |
| D308N -F | GCACCGGATACGGAGAA**AAC**TGGCATCCAAGTATTGCCACCC |
| D308N -R | GGGTGGCAATACTTGGATGCCA**GTT**TTCTCCGTATCCGGTGC |
| Y184A -F | CGGACTTACCATGAAC**GCG**GGCGGAGCCCCCGG |
| Y184A -R | CCGGGGGCTCCGCC**CGC**GTTCATGGTAAGTCCG |
| W265A -F | CGGTCCGATGCTT**GCG**GGAACGGGCCTGGATTTGTGCC |
| W265A -R | GGCACAAATCCAGGCCCGTTCC**CGC**AAGCATCGGACCG |
| W309A -F | CCGGATACGGAGAAGAC**GCG**CATCCAAGTATTGCCACCC |
| W309A -R | GGGTGGCAATACTTGGATG**CGC**GTCTTCTCCGTATCCGG |
